# NOS1 *S*-nitrosylates PTEN and inhibits autophagy in nasopharyngeal carcinoma cells

**DOI:** 10.1038/cddiscovery.2017.11

**Published:** 2017-02-20

**Authors:** Lingqun Zhu, Linlin Li, Qianbing Zhang, Xiao Yang, Zhiwei Zou, Bingtao Hao, Francesco M Marincola, Zhengjun Liu, Zhuo Zhong, Meng Wang, Xiaoxuan Li, Qianli Wang, Keyi Li, Wenwen Gao, Kaitai Yao, Qiuzhen Liu

**Affiliations:** 1 Cancer Research Institute, Southern Medical University, Guangzhou, China; 2 Sidra Medical and Research Center, Out-Patient Clinic, PO Box 26999, Al Luqta Street, Education City North Campus, Qatar Foundation, Doha, Qatar; 3 Department of Vascular Surgery, Nanfang Hospital Southern Medical University, Guangzhou, China

## Abstract

Autophagy is a cellular survival mechanism that involves the catabolic degradation of damaged proteins and organelles during periods of metabolic stress, and when overly stimulated, commonly contributes to cell death. Nitric oxide (NO), a potent cellular messenger, participates in a complex mechanism which assists in controlling autophagy. However, the mechanism by which endogenous NO formed by distinct isoforms of nitric oxide synthase (NOS) helps to regulate autophagy in cancer cells remains unclear. Here we report that NOS1 reduces excessive levels of autophagy and promotes the survival of nasopharyngeal carcinoma cells. We found that inhibition of NOS1 increased cell death resulting from siRNA or the use of pharmacologic agents; and this effect was reversed by the autophagy inhibitor, chloroquine. The role of NOS1 in the autophagy process depended on the activation of AKT/mTOR signaling by *S*-nitrosylation of phosphatase and tensin homolog (PTEN) proteins. The mechanism by which NOS1 modifies PTEN protein might involve a direct interaction between these two molecules. Moreover, in an *in vivo* study, the NOS1 inhibitor N(G)-nitro-L-arginine methyl ester activated AKT/mTOR signaling and promoted autophagy in xenograph tumors. Our studies demonstrated that NOS1 prevents excessive autophagy via *S*-nitrosylation of PTEN, and activation of the AKT/mTOR signaling pathway. PTEN and the AKT/mTOR signaling pathway are promising targets for improving the chemotherapeutic treatment of cancer.

## Introduction

Autophagy is a cellular self-digestion mechanism involving the catabolic degradation of damaged proteins and organelles during periods of metabolic stress, and is required for tumor cell survival during periods of starvation and ongoing tumorigenesis.^1,2^ However, excessive autophagy is commonly associated with cell death,^3^ and thus autophagy must be carefully regulated if cells are to survive under stressful conditions. Targeting the pro-death and pro-survival functions of autophagy has become a novel therapeutic strategy for treating cancer.^4,5^ Nitric oxide (NO) is a ubiquitous messenger molecule capable of regulating multiple cellular signaling pathways,^6,7^ including those which modulate autophagy.^8,9^ Exogenous NO produced by the NO donor compound DETA-NONOate or the overexpression of three NOS family members (NOS1, NOS2, and NOS3) creates a decrease in autophagic flux.^8^ Moreover, exogenous NO can induce *S*-nitrosylation of JNK1 and IKKB proteins,^10^ and thereby regulate autophagy via mTOR-dependent and independent mechanisms. *S*-nitrosylation of IKKB reduces AMPK phosphorylation, leading to mTORC1 activation via TSC2 (IKKB-AMPK-TSC2). Furthermore, *S*-nitrosylation of JNK1 reduces Bcl-2 phosphorylation; resulting in increased formation of the Bcl-2-Beclin1 complex (JNK-BCL-2-Beclin1).^8^ As inhibiting the function of endogenous NOSs by L-NAME-enhanced autophagy does not require the two pathways mentioned above, endogenous NOSs in their basal state might exert their effects by other mechanisms.^8^ NO and nitric oxide synthases are ubiquitous enzymes in malignant tumors, and known to exert both pro- and anti-tumor effects.^11^ When present at low to intermediate concentrations, NO stimulates various oncogenic signaling pathways such as AKT, ERK, and HIF, and when present at high concentrations, NO can produce nitrosative stress and stimulate anti-oncogenic signaling mechanisms such as the p53 and apoptosis pathways.^7,11,12^ Endogenous NO is formed by three NOS isoforms (NOS1, NOS2, and NOS3) that differ in the way they modify transcription processes and enzyme activity.^6,7^ NOS1 and NOS3 are constitutively expressed in cells, and both produce low levels of NO in response to stimulation of intracellular cellular calcium flux. In contrast, NOS2 is inducible, becomes increasingly expressed in response to inflammation, and produces high levels of NO in a calcium-independent manner.^7^ Thus, the existing evidence suggests that NOS1 and NOS3 mainly function in promoting tumor development, while depending on the state of a cell, NOS2 might also play an additional role in tumor progression, However, it remains unknown whether distinct NOS isoforms play different roles in regulating autophagy, and which signaling pathways are involved.

The mammalian target of rapamycin (mTOR) is a critical regulator of autophagy.^13^ However, the mTOR signaling pathway is regulated by numerous other upstream signaling pathways including PI3K/Akt, AMPK, and p53. In particular, the mTOR pathway is activated when levels of growth factors or amino acids become sufficiently low to create a cellular energy crisis, and in this manner, modulates autophagy based on the cellular energy requirements.^14^ PTEN, a dual protein/lipid phosphatase, is a key regulator of the AKT/mTOR pathway. A mutation in the *PTEN* gene or a downregulation of PTEN protein production are frequent occurrences in several types of cancer,^15,16^ and lead to activation of the AKT/mTOR signaling pathway, which is associated with a poor clinical prognosis. Recently, PTEN in neuron cells was reported to become selectively *S*-nitrosylated at cysteine residue (Cys-83) in the presence of low concentrations of NO.^17^ However, it is unclear whether endogenous NOS plays a role in inducing *S*-nitrosylation of PTEN to form SNO-PTEN. Here we report that NOS1 induces *S*-nitrosylation of PTEN. This leads to increased Akt/mTOR signaling activity, which results in inhibition of autophagy and promotion of cell viability.

## Results

### Exogenous NO plays a dual role in regulating autophagy in NPC cells

**Figure 1:**
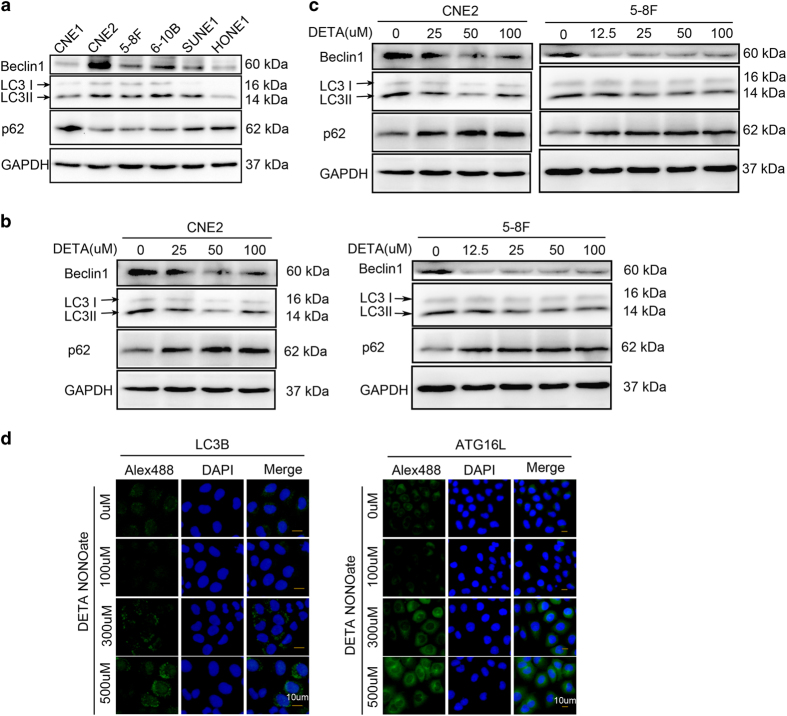
Exogenous NO plays a dual role in regulation of autophagy. (**a**) Basic level of autophagy in six of NPC cell lines was tested by western blot using antibodies against LC3B, Beclin1 (autophagy-related protein) and p62 (autophagy substrate). (**b**) Immunoblot shows that autophagy was inhibited by low concentration of exogenous NO (DETA-NONOate 0–100 *μ*M for 24 h) in CNE2 (left) and 5–8F (right). (**c**) Immunoblot shows that autophagy was increased by high concentration of exogenous NO (DETA NONOate 125–500 *μ*M for 24 h) in CNE2 (left) and 5–8F (right). (**d**) Representative images by inversed fluorescent microscope showed that LC3B (left) and ATG16L1 (right) were inhibited by low dose but increased by high dose of NO after immunostained with primary antibodies and corresponding FITC-conjugated secondary antibodies. Nuclei were counterstained with DAPI. Representative images of each sample are shown. (Blots were probed for GAPDH as a control for equal protein loading in all lanes).

We first evaluated the basic level of autophagy in six NPC cell lines (CNE1, CNE2, 6-10B, 5-8F, SUNE1, and HONE1) by immunoblotting with antibodies against autophagy proteins LC3-II and Beclin1, and autophagy substrate, p62. Among the six cell lines evaluated, CNE2 and 5-8F are more aggressive cell lines when compared with CNE1 and 6-10B, respectively.^18,19^ Immunoblotting studies revealed that autophagy-related proteins LC3-II and Beclin1 were highly expressed in CNE2 and 5-8F cells, moderately expressed in 6-10B and SUNE1 cells, and only slightly expressed in CNE1 and HUNE1 cells, while the autophagy substrate p62/SQSTM1 showed an opposite trend of expression (Figure 1a). This result suggested that more aggressive NPC cell lines have higher levels of autophagy. Therefore, we decided to use CNE2 and 5-8F cells with high levels of autophagy in our subsequent experiments investigating the effects of NO on autophagy.

The effect of NO on cellular functions depends on the length of exposure and the NO concentration.^11^ The physiological concentration of NO released by endogenous NOS isoforms is <300 nM. We treated CNE2 and 5-8F cells with different concentrations of the NO donor DETA NONOate (0–500 *μ*M) to sustain a certain NO concentration range (5–200 nM) in the cell culture medium. Immunoblotting studies revealed that CNE2 and 5-8F cells cultured with low concentrations of DETA NONOate (12.5–100 umol/l) expressed lower amounts of Beclin1 protein, but higher amounts of p62; additionally, their cellular levels of LC3-II protein were less obviously reduced (Figure 1b). Interestingly, these trends were reversed in cells cultured with high concentrations (>100 *μ*M) of DETA NONOate (Figure 1c). Immunofluorescence staining showed that the amount of LC3-II protein (green fluorescence spots) in the cytoplasm of CNE2 cells was reduced by high levels of NO (Figure 1d). These data indicate that low concentrations of NO decrease autophagy levels, while higher NO concentrations increase autophagy. Moreover, immunofluorescence staining also showed that the levels of Atg16L1, an early autophagy-related protein, were regulated in a bidirectional manner by different concentrations of NO (Figure 1d). Thus, NO appears to help regulate autophagy during its early stage of flux.

### NOS1 inhibits autophagy in NPC cells

**Figure 2:**
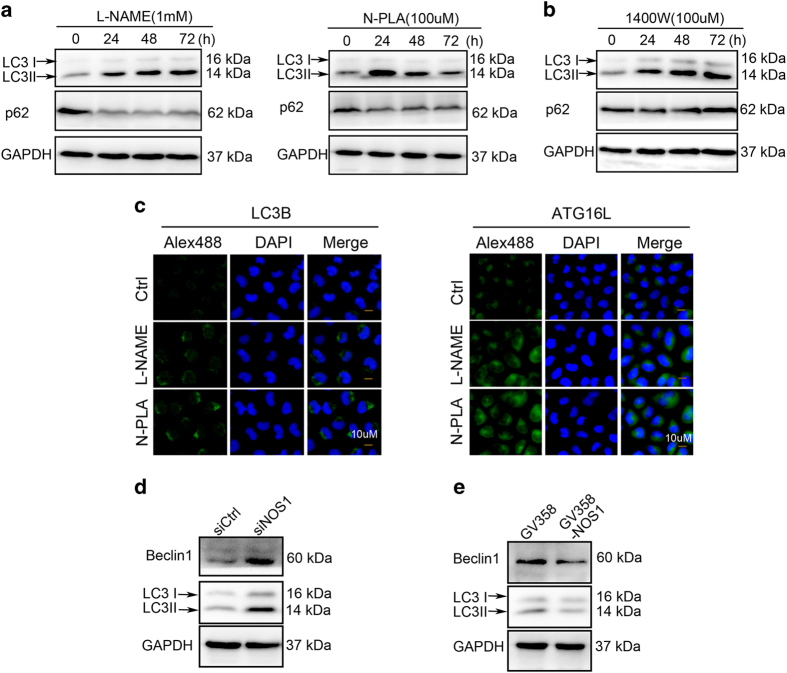
NOS1 activity and expression in NPC cells control the base level of autophagy. (**a**) NO of CNE2 cells could be reduced by treatment with any of the three inhibitors. The relative amount of NO released into the culture medium was tested by the Griess method after treated with L-NAME (1 mM), N-PLA (100 *μ*M), or 1400W (100 *μ*M), respectively, in CNE2 cells for 24 h. (**b**) Inhibition of NOS1 activity by non-selective inhibitor L-NAME (left) or selective inhibitor N-PLA (right) for various time periods (24, 48, and 72 h) increased autophagy level CNE2; inhibition of NOS2 activity with selective inhibitor 1400W increased LC3B but not decreased substrate p62 tested by western blot. (**c**) Representative images of increased LC3B (left) and ATG16L1 (right) detected by immunofluorescent after treated with L-NAME and 1400W for 24 h (nuclei were counterstained with DAPI). (**d**) Downregulation of NOS1 by siRNA induced increased autophagy level in CNE2 cells. (**e**) NOS1 overexpression after infected with GV358-NOS1 for 48 h decreased the level of autophagy in CNE2 cells. GAPDH was used as a loading control.

The three isoforms of NOS are known to release different amounts of NO *in vivo*.^7^ We had previously evaluated the amount of NO released by cancer cells grown in our culture medium, and found that the concentration of NO released by cancer cells in our culture medium was 10% of that released by cells which had been treated with 100 *μ*M DETA-NONOate (~40 nM NO) under the same conditions.^20,21^ This concentration of NO is much lower than that released by NOS2 under conditions of inflammatory stimulation. To evaluate the relative amount of NO released into the culture medium by each of the NOS isoforms, we treated NPC cells with either a broad-spectrum NOS inhibitor (L-NAME),^22^ or a selective NOS1 or NOS2 inhibitor (N-PLA or 1400W, respectively). We found that the amount of NO formed by CNE2 cells could be reduced by treatment with any of the three inhibitors (L-NAME, N-PLA, or 1400W). Treatment with L-NAME produced a greater reduction in the amount of NO formed by CNE2 cells when compared to treatment with either N-PLA or 1400W. Moreover, treatment with N-PLA produced less of a reduction in NO concentration when compared to treatment with 1400W. These data suggest that while all three NOS isoforms contribute to the amount of NO released into culture medium, NOS1 may produce less NO when compared with NOS2 (Figure 2a).

To assess the role of each distinct NOS isoform in regulating autophagy, we evaluated the effect of individual NOS inhibitors on expression of LC3B and p62 proteins in CNE2 cells. Immunoblotting studies revealed that LC3B expression was increased, but p62 expression was decreased in CNE2 cells treated with L-NAME or N-PLA for 48 h; while treatment with 1400W increased LC3B expression, and did not reduce p62 expression (Figure 2b). Additionally, immunofluorescence assays showed that inhibition of NOS1 with either L-NAME or N-PLA increased the punctate fluorescence that indicates LC3B and ATG16L1 expression in the cytoplasm of CNE2 cells (Figure 2c). To verify NOS1 as an inhibitor of autophagy in NPC cells, we evaluated any changes in autophagy that occurred following *NOS1* knockdown and overexpression, respectively. As expected, expression of LC3B and Beclin1 proteins was markedly increased in CNE2 cells containing siNOS1; however, both proteins showed markedly decreased expression in CNE2 cells which overexpressed NOS1, when compared with expression in their control groups (Figures 2d and e). These results suggest that under basal conditions, NOS1 helps to inhibit autophagy by producing a small amount of NO. In contrast, NOS2 only partially impacts the formation of autophagosomes during the early phase, and is not an effective inhibitor of autophagy.

### Autophagy inhibition by NOS1 contributed to cell survival and resistance to cisplatin (DDP) in NPC cells

**Figure 3:**
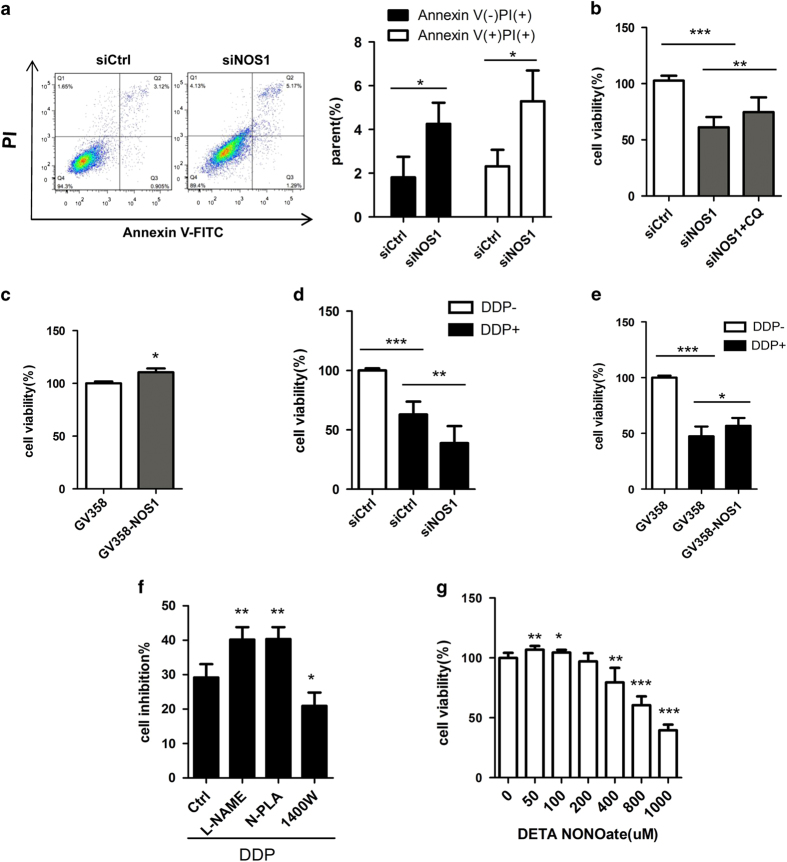
Autophagy inhibition by NOS1 contributed to cell survival and chemoresistance to DDP in NPC cells. (**a**) Flow cytometry analysis indicates siNOS1 increased the death (PI positive ) and apoptosis (Annexin V positive) cells in CNE2; (**b**) The increased cell death by NOS1 siRNA was reversed significantly by autophagy inhibitor chloroquine in CNE2 compared to siRNA control tested by MTT or flowcytometry. (**c**) Overexpression of NOS1 by transfer with GV358-NOS1 promoted cell growth in CNE2 cells tested with MTT. (**d**, **e**) siNOS1 (**d**) increased while NOS1 overexpression (**e**) decreased the cell death induced by treatment with 2.5 *μ*M DDP (DDP) for 48 h measured by MTT assay as compared to control. (**f**) Treated with selective inhibitor for NOS1 (N-PLA) but not for NOS2 (1400W) increases the sensitivity of CNE2 to DDP treatment as measured by MTT assay. (**g**) Cells were incubated with the indicated doses of DETA NONOate for 48 h, cell viability was analyzed by MTT. (The figure showed by combining the value of three independent experiments; values=mean±S.E., *P<0.05; **P<0.01; ***P<0.001).

Cancer cells employ increased rates of autophagy as a survival mechanism to protect themselves against different types of cellular stress.^1^ Flow cytometry studies revealed that *NOS1* knockdown by siRNA significantly increased both the PI and Annexin V-FITC positive cell populations, which are indicative of dead cells and apoptotic cells, respectively (Figure 3a). We performed *NOS1* knockdown and overexpression studies to investigate how NOS1 might affect the viability of NPC cells. MTT assays showed that when compared with the control groups, knockdown of *NOS1* greatly reduced the numbers of viable NPC cells, while overexpression of NOS1 did not have this effect. Moreover, CNE2 cells that overexpressed NOS1 displayed increased proliferation after 48 h (Figures 3b and c). We next used the autophagy inhibitor chloroquine to investigate whether the increased cell death seen among *NOS1* knockdown cells was related to autophagy. MTT tests showed that the increased cell death which occurred following *NOS1* knockdown could be partially blocked by treatment with chloroquine (Figure 3b). This result suggests that NOS1 promotes cell viability *in vitro* by regulating autophagy.

A previous study showed that depletion of endogenous NO enhanced DDP-induced cell death.^23^ To examine whether NOS1 might play a role in chemotherapy resistance, we investigated the effect of genetically knocking down or forcing overexpression of *NOS1* on the viability of CNE2 cells being treated with DDP. Following DDP treatment, cells with siNOS1 were significantly less viable when compared with control cells (Figure 3d). In contrast, overexpression of NOS1 partially blocked DDP-induced cell death (Figure 3e). We next evaluated whether inhibiting NOS1 could improve the effect of chemotherapy. Cells were treated with DDP in the presence or absence of a single NOS inhibitor (either L-NAME, N-PLA, or 1400W). MTT assays showed that NOS1 inhibition caused by either L-NAME or N-PLA increased the level of cell death induced by DDP, while NOS2 inhibition caused by 1400W protected cells from the effects of DDP treatment (Figure 3f). We also used different doses of the NO-releasing compound DETA NONOate to test whether exogenous NO might regulate NPC cell viability. We found that while low concentrations (<100 *μ*M) of DETA NONOate could mildly increase cell viability, its effect on viability was not as significant as that produced by NOS1 overexpression. Moreover, high concentrations of artificially released NO reduced the percentage of viable cells (Figure 3g). The increased viability of CNE2 cells exposed to a low concentration of exogenous NO suggests that NOS1 regulates cell viability through its products of NO. The results described above indicate that NOS1 inhibits autophagy, contributes to cell viability, and plays a role in the chemoresistance of NPC cells. Additionally, inhibition of NOS1 either by genetic or pharmacologic methods promotes DDP-induced cell death.

### Autophagy inhibition by NOS1 depends on activation of the AKT/mTOR pathway

**Figure 4:**
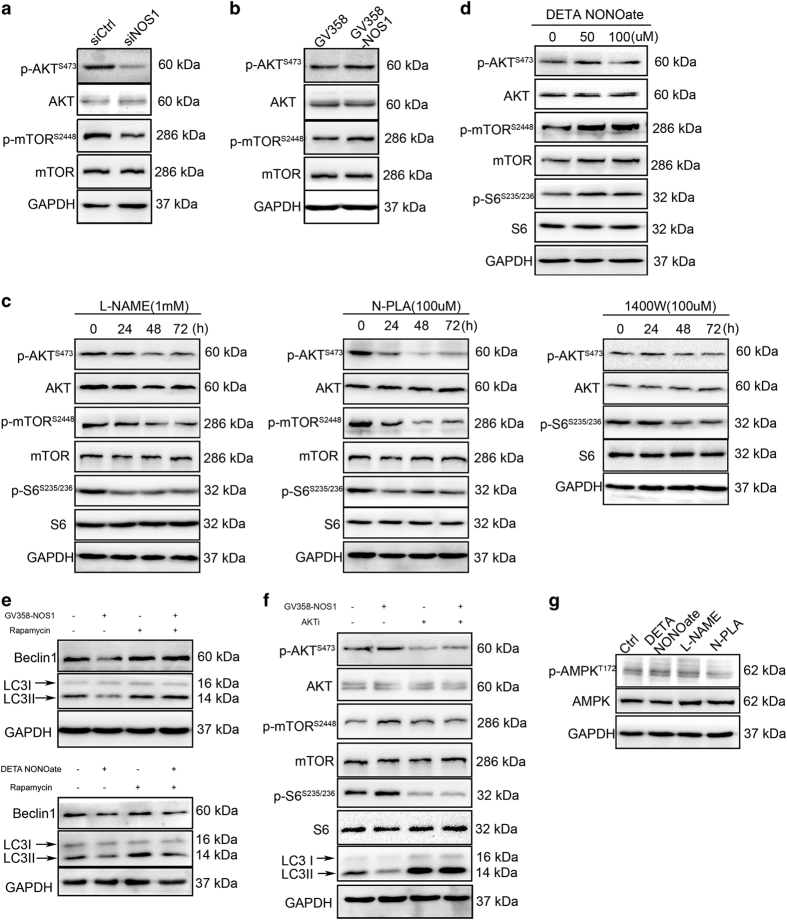
NOS1 inhibition on autophagy depends on the activation of AKT/mTOR signal in NPC cells. (**a**, **b**) siRNA-NOS1 (**a**) decreased while overexpression-NOS1 (**b**) increased the p-AKT, p-mTOR, and p-S6 level. (**c**) Treated with L-NAME (1 mM, left) or N-PLA (100 *μ*M, median) but not 1400W (right) decreased the level of p-AKT, p-mTOR, and p-S6 tested by immunoblotting with antibody for p-AKT(Ser473), AKT, p-mTOR (Ser2448), mTOR, p-S6 (S235/236), and S6. (**d**) Treated with either DETA NONOate by indicated concentration for 24 h increased the p-AKT, p-mTOR, and p-S6 level in CNE2 tested by western blot. (**e**) Treated with mTOR inhibitor rapamycin for 24 h reversed overexpresison NOS1 (up) or NO donor (down) induced the increasing of autophagy. (**f**) Inhibition of AKT with AKT inhibitor II for 24 h increased the autophagy level, which had been inhibited by being treated with NOS1 overexpression tested by immunoblotting with antibody for autophagy protein and events of AKT/mTOR signal. (**g**) Treated with 50 *μ*M DETA-NONOate or NOS1 inhibitor L-NAME (1 mM) or N-PLA (100 *μ*M) did not alter the level of p-AMPK*α* (T172) and AMPK*α* by immunoblotting; GAPDH was probed to ensure equal protein loading.

The AKT/mTOR signaling pathway is constitutively activated in nasopharyngeal carcinoma (NPC) tissue.^24^ To investigate whether the role played by NOS1 in autophagy inhibition depends on activation of AKT/mTOR signaling, we evaluated the effect of NOS1 on AKT/mTOR signaling induced by NOS1 siRNA, as well as by overexpression of NOS1. The activity status of the AKT and mTOR pathways was evaluated by determining the levels of phosphorylated AKT and TOR proteins (p-AKT and p-mTOR, respectively). Immunoblotting studies showed that the levels of p-AKT and p-mTOR were decreased by *NOS1* knockdown with siRNA, while the total amounts of AKT and mTOR proteins remained unchanged. However, slightly increased levels of p-AKT and p-mTOR proteins were detected when NOS1 was overexpressed (Figures 4a and b). To investigate whether only NOS1 activates AKT/mTOR signaling, we evaluated the effects of several NOS inhibitors (L-NAME, N-PLA, and 1400W) on the activation of AKT/mTOR signaling. A 48-h treatment with either L-NAME or N-PLA significantly reduced the levels of p-AKT and p-mTOR proteins, as well as the downstream levels of phosphorylated mTOR S6 (p-S6). In contrast, treatment with 1400W did not significantly reduce the levels of p-AKT, but did significantly reduce the levels of p-S6 (Figure 4c). Moreover, exogenous NO produced by 50–100 *μ*M DETA NONOate significantly increased the levels of phosphorylated S6 present in CNE2 cells after 48 h of treatment, but only slightly increased the levels of p-AKT (Figure 4d). These data indicate that the ability of NOS1 to activate mTOR is dependent on AKT activation, while NOS2 or a certain level of NO might activate mTOR signaling via another mechanism upstream of mTOR, instead of via the AKT pathway.

Next, we used chemical inhibitors of AKT and mTOR (Akt1/2 kinase inhibitor and rapamycin, respectively) to investigate whether NOS1-induced autophagy inhibition was correlated with the role of NOS1 in activating the AKT/mTOR pathway. Treatment with rapamycin not only increased the basal levels of LC3B and Beclin1 in CNE2 cells, but also reversed the decreases in LC3B and Beclin1 levels which had been induced by treatment with DEAT-NONOate or by NOS1 overexpression (GV358-NOS1; Figure 4e). Treatment with Akt1/2 kinase inhibitor significantly decreased the basal levels of p-AKT, and markedly reversed the increased p-AKT levels which had been induced by NOS1 overexpression. Additionally, Akt1/2 kinase inhibitor treatment also decreased the levels of p-S6 and increased the levels of LC3B expression (Figure 4f). These results demonstrated that NOS1-induced activation of mTOR signaling and the upstream AKT pathway is indispensable for the ability of NOS1 to reduce autophagy inhibition.

A previous study showed that AMPK activates autophagy by inhibiting mTOR and directly phosphorylating ULK1.^25^ To determine whether regulation of mTOR by NOS1 in NPC cells is dependent on AMPK activity, we used western blotting to detect the levels of p-AMPK and AMPK proteins in NPC cells treated with a NOS1 inhibitor or NO donor. We found no dramatic difference regarding the results obtained in the presence or absence of a NO donor and NOS1 inhibitor (Figure 4g). This result suggests that activation of mTOR by NOS1 does not require participation of the AMPK pathway. Taken together, these results indicate that inhibition of autophagy proteins in NPC cells is dependent on AKT/mTOR signaling.

### NOS1 activates AKT/mTOR signaling by inducing *S*-nitrosylation of PTEN

**Figure 5:**
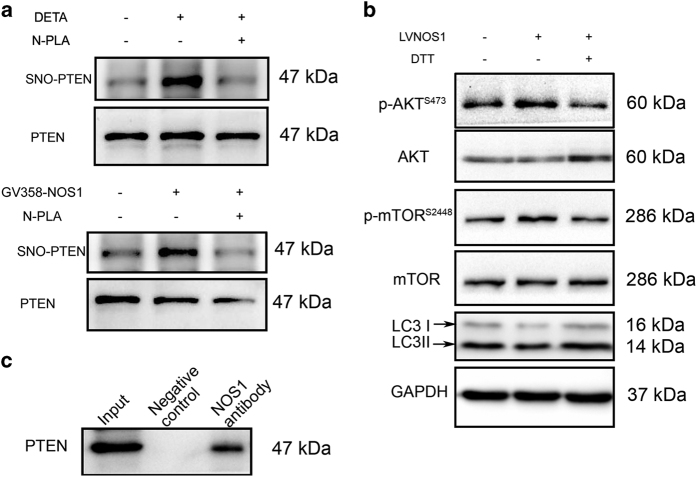
NOS1 activates AKT/mTOR pathway through inducing *S*-nitrosylation on PTEN. (**a**) Treated with 50 *μ*M DETA-NONOate (up) or infected with GV358-NOS1 (down) for 24 h increased the level of *S*-nitrosylation PTEN as tested by biotin-switch assay with antibody for *S*-nitrosylation in CNE2 cells. Total PTEN in cell lysates was loaded as control. (**b**) De-*S*-nitrosylation of PTEN with reducing agent 1,4-Dithiothreitol (500 *μ*M) for 24 h reversed the increased p-AKT and p-mTOR induced by NO donor or overexpresion NOS1 as detected by western blot. (**c**) Interaction of NOS1 with PTEN in NOS1-GV358 cells were detected by co-immunoprecipitated with anti-NOS1 antibody. Western blotting was performed by using indicated antibody.

PTEN is a crucial negative regulator of AKT/mTOR signaling, and in neural cells, is modulated by NO-induced *S*-nitrosylation.^17^ We investigated whether NOS1 nitrosylates PTEN to activate AKT/mTOR signaling. The levels of *S*-nitrosylated PTEN in CNE2 cells were measured by immunoblotting with an *S*-nitrosylation antibody after the cells had been treated with 50 *μ*M DETA NONOate or had overexpressed NOS1. Our results showed that both exogenous NO produced by DETA NONOate and endogenous NO produced by NOS1 overexpression significantly enhanced the levels of *S*-nitrosylated PTEN, as compared with those levels found in control cells (Figure 5a). The increased *S*-nitrosylation of PTEN was followed by an enhanced phosphorylation of AKT and S6 proteins in the AKT/mTOR pathway (Figure 5b). Moreover, depletion of *S*-nitrosylation activity by treatment with the reducing agent 1,4-Dithiothreitol reversed the increased levels of p-AKT and p-S6 produced by NOS1 overexpression. This reversal was followed by a recovery of autophagy-related protein expression (Figure 5b). These data suggest that NOS1-induced SNO-PTEN promoted activation of the AKT/mTOR signaling pathway in NPC cells.

Although NO can be toxic to cells, it has a very short biological half-life.^6^ NOS1 activity must be tightly regulated by targeting its activity toward specific subcellular sites via PDZ domains, which are essential for establishing functional protein networks that control diverse cellular functions.^26,27^ PTEN possesses a C-terminal PDZ-binding motif that is recognized by a specific set of PDZ domains found in scaffolding and regulatory proteins or enzymes.^28,29^ In some instances, stabilization of PTEN protein increases its catalytic activity, as measured by decreased p-AKT levels.^30,31^ We investigated whether NOS1 binds to PTEN for purposes of nitrosylating PTEN. Co-immunoprecipitation assays showed that NOS1 detected by an anti-NOS1 antibody in GV358-NOS1 cells co-immunoprecipitated with PTEN (Figure 5c).

### NOS1 inhibits autophagy *in vivo* by activating PTEN/AKT/mTOR signaling

**Figure 6:**
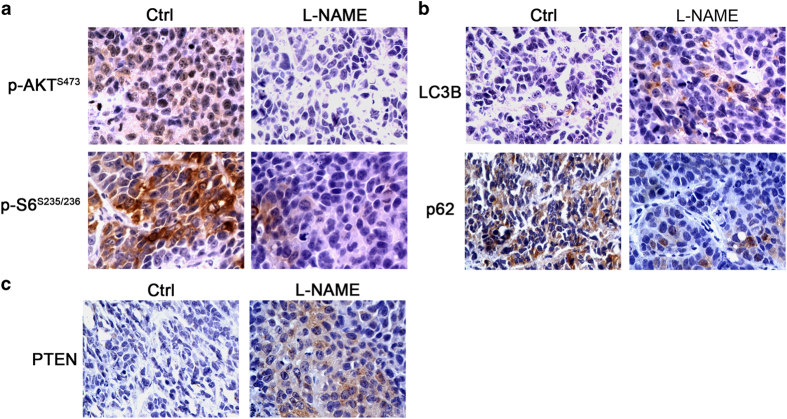
L-NAME decreased the activation of AKT/mTOR signal and increased autophagy level *in vivo*. (**a**) Representative images of immunohistochemistry analysis show that L-NAME decreased the p-AKT and p-S6 in xenograph tumor. (**b**) Representative images of immunohistochemistry analysis show that L-NAME increased the expression of autophagy protein LC3B and autophagy substrate p62. (**c**) Representative images of immunohistochemistry analysis show that L-NAME also increased the expression of PTEN mildly. All photos were taken under a microscope at ×400 magnification.

In our previous study using a NPC xenograft model with CNE2 cells, we observed that inhibition of NOS by treatment with L-NAME resulted in markedly inhibited tumor growth (data not shown). To verify that NOS1 inhibits autophagy *in vivo*, we performed histochemical analyses that evaluated the effect of L-NAME treatment on the level of autophagy and activation of AKT/mTOR signaling in both control and L-NAME-treated samples. The results showed that L-NAME inhibited these processes at the level of p-AKT and p-S6 (Figure 6a). Moreover, L-NAME upregulated expression of autophagy-related protein LC3B and downregulated the autophagy substrate, p62 (Figure 6b).

*S*-nitrosylation of PTEN facilitates its ubiquitin-mediated degradation, therefore, we investigated the level of PTEN protein in tissue affected by NOS inhibition. Immunohistochemistry studies performed with a PTEN antibody indicated that treatment with L-NAME decreased the expression of PTEN when compared with its expression in a control group. Interestingly, NOS1 expression was also reduced by L-NAME. The possible existence of a back loop in NOS1-mediated PTEN regulation needs to be investigated in future experiments. When taken together, our data consistently support our conclusion that NOS1-mediated inhibition of autophagy in NPC cells depends on *S*-nitrosylation of PTEN, which mediates the activation of AKT/mTOR signaling.

## Discussion

In this study, we demonstrated that a small amount of NO produced by NOS1 was capable of inducing *S*-nitrosylation of PTEN. This led to activation of the AKT/mTOR signaling pathway, the downregulation of autophagy, and a resultant increase in cell survival. Our results imply that inhibition of NOS1 by either genetic or pharmacologic methods can inhibit the AKT/mTOR pathway and decrease chemoresistance displayed by cancer cells. This information may provide a clue for managing chemoresistance in cases of NPC.

NPC is common throughout southern China, and has a 25-fold greater incidence in this region when compared with its incidence in other countries. Moreover, NPC has been consistently associated with the Epstein–Barr virus.^32^ The PI3K-AKT-mTOR signaling pathway is frequently activated in cases of NPC, and this activation is critical for cancer cell survival; however, it is also correlated with a poor clinical prognosis.^24^ The EBV latent membrane proteins LMP1 and LMP2A are capable of inactivating PTEN,^33^ which is an important tumor suppressor protein in various types of cancer cells. Previous studies have shown that PTEN phosphatase negatively regulates PI3K/Akt signaling,^34,35^ but is frequently inactivated by a gene deletion or mutation, and also degraded in the proteosomes of many types of cancer cells.^36^ Various types of post-translational modifications, including phosphorylation, oxidation, and acetylation, are also known to regulate the function and stability of PTEN.^37^ In this study, we confirmed that endogenous NOS could mediate *S*-nitrosylation of PTEN and activate AKT/mTOR signaling, resulting in decreased levels of autophagy and increased chemoresistance.

Protein *S*-nitrosylation is a covalent post-translational modification that results from coupling a NO moiety containing a reactive thiol group to a protein cysteine residue to form an *S*-nitrosothiol (SNO) moiety.^38^ *S*-nitrosylation plays a key role in the transmission of NO-based cellular signals involved in vital cellular processes, including transcription regulation, DNA repair, apoptosis, and autophagy.^10,38,39^ Low concentrations of either exogenous or endogenous NO can selectively induce *S*-nitrosylation of PTEN at a specific cysteine residue (Cys-83).^17^ Moreover, *S*-nitrosylation of PTEN leads to degradation of PTEN via a ubiquitin ligase NEDD4-1-mediated mechanism involving ubiquitin. This degradation is followed by hyper-activation of the Akt cascade in neuron cells.^40^ In this study, we showed that NOS1 induces *S*-nitrosylation of PTEN in NPC cells, leading to downregulation of PTEN expression and activation of the downstream AKT/mTOR pathway. This finding provides a new mechanism for the dysregulation of AKT/mTOR signaling in NPC cells, and suggests a new target that might be exploited when attempting to manage chemoresistance by regulating autophagy.

Although all three NOSs (NOS1, NOS2, and NOS3) exist in NPC cells and contribute to the total NO concentration in NPC cells, only NOS1 was shown to activate AKT/mTOR signaling by *S*-nitrosylation of PTEN. NOS2 produced higher levels of NO in CNE2 cells, it only slightly *S*-nitrosylated PTEN. One possible factor underlying the special ability of NOS1 to nitrosylate PTEN might be the unique PDZ domain possessed by NOS1.^26^ A PDZ domain consists of ~90 amino acids. Modular interactions mediated by PDZ domains facilitate the selective and effective interaction of NOS1 with its target substrate. In brain tissue, NOS1 is targeted toward synaptic membranes by its interactions with scaffolding proteins PSD-95 and PSD93, which anchor two PDZ domains.^41^ NOS1 interacts with its substrate by recognizing and selectively binding the specific C terminus of its target protein molecule via the PDZ domain. PTEN possesses a C-terminal PDZ-binding motif that is recognized by a specific set of PDZ domains found in scaffolding and regulatory proteins. ^29^ Many of the proteins that interact with PTEN via PDZ domains are multi-PDZ-domain scaffolding proteins that stabilize PTEN and decrease p-AKT levels.^42^ However, the binding of PTEN to a specific PDZ domain containing NOS1 has not yet been reported. For the first time, this study verified that NOS1 selectively modifies PTEN by *S*-nitrosylation, which is dependent on its interaction with PTEN. Future studies will verify whether NOS1 directly or indirectly interacts with PTEN via scaffolding and regulatory proteins containing PDZ domains. Our current findings help to elucidate the effects of NOS1 on autophagy, and provide a detailed molecular mechanism explaining the efficacy of NOS1 in modulating autophagy. The affects produced by NOS1 contribute to cell survival and impact the development of chemoresistance. As a result, our findings may provide clues for improving the treatment of NPC.

## Materials and Methods

### Cell lines and culture

The human NPC cell lines, CNE1, CNE2, 6-10B, 5-8F, SUNE1, and HONE1, were from previous storage cells of our institute. Cells were maintained in RPMI 1640 (Invitrogen, Gibco, China) supplemented with 10% fetal bovine serum (FBS; BI, Salt Lake City, UT, USA) in a humidified 5% CO_2_ atmosphere at 37 °C. Stable NOS1 overexpression and nontargeted control cell lines were generated by CNE2 cells that infected with lentivirus vector GV358 (Ubi-MCS-3FLAG-SV40-EGFP-IRES-puromycin) encoding NOS1 or control (Genechem, Shanghai, China) according to the manufacturer’s instructions. Stable clones were infected with lentivirus and selected in culture medium containing puromycin (2 *μ*g/ml).

### Antibodies and reagents

DETA NONOate(DETA), L-NAME, N-PLA, 1400W, and the Griess agents were purchased from Cayman Chemical (Ann Arbor, MI, USA). Chloroquine diphosphate salt, Akt1/2 kinase inhibitor, DDP, 1,4-Dithiothreitol, and thiazolyl blue tetrazolium bromide (MTT) were purchased from Sigma-Aldrich (St Louis, MO, USA), rapamycin was purchased from LC Laboratories (Woburn, MA, USA). The primary antibody against GAPDH and *β*-actin was purchased from Sigma-Aldrich. Antibodies recognizing LC3B, p-AKT(Ser473), AKT, p-mTOR (Ser2448), mTOR, p-S6 (S235/236), S6, and p-AMPK*α* (Thr172) were purchased from Cell Signaling Technology (Beverly, MA, USA). Antibodies against Beclin1, p62, NOS1, PTEN, and AMPK were obtained from Abcam (Cambridge, MA, USA). Atg16 L were purchased from MBL (Nagoya, Japan). Secondary antibody peroxidase conjugated goat anti-rabbit IgG (h+l) and peroxidase conjugated goat anti-mouse IgG (h+l) were purchased from fdbiotech (Guangzhou, China). Goat anti-rabbit Alexa Fluor 488and DAPI were purchased from Invitrogen (Carlsbad, CA, USA).

### Small interfering RNAs

The target sequence for NOS1 siRNA was 5′-CTAGCACTTACCAGCTCAA-3′, the control siRNA sequence was chemically synthesized by RiboBio Co. Ltd. (Guangzhou, China). Cells were seeded into six-well plates the day before transfection. Thereafter, cells were transfected with serum-free Opti-MEM media (Invitrogen, Gibco, China), lipofectamine 2000 (Invitrogen), and 100 nM siRNA according to the manufacturer’s recommendations. The medium was replaced with fresh growth medium containing 10% fetal bovine serum 6 h after transfection, and cells were incubated for an additional 24 h for subsequent experiments.

### Immunoblotting

Cells were plated into six-well plate 1 day before indicated treatment and were washed twice with cold PBS before added in RIPA buffer with 1 mM PMSF and 1 mM phosphatase inhibitor mixture. The protein concentration in each sample was determined using the Bradford assay. After normalization, equal amounts of proteins were fractionated on 8–15% SDS-PAGE gels. The proteins were then transferred to PVDF membranes (Millipore, Boston, MA, USA) and incubated with the indicated primary antibodies and corresponding HRP-conjugated secondary antibodies. The immunoreactive bands were visualized by chemiluminescence according to the manufacturer’s recommendations (Millipore).

### Immunofluorescence

Immunofluorescence was performed as previously described.^8^ For immunofluorescent staining, the cells were fixed with 4% paraformaldehyde after indicated treatment. After incubation with primary antibody, included LC3B and ATG16L, the slides were incubated with Alexa Fluor 488-conjugated secondary antibody for 1 h, followed by counterstaining with DAPI solution. Images were acquired using a Nikon Eclipse TE2000 Inverted Fluorescence Microscope System (Nikon, Tokyo, Japan) and analyzed using Nikon software. Representative images are shown in figures with identical settings.

### Co-immunoprecipitation

Immunoprecipitates were obtained using Pierce Co-Immunoprecipitation (Co-IP) Kit (Cat. No.26149). The assay was performed according to the manufacturer’s instructions. Using this method enables isolation of native protein complexes from cells lysate by directly immobilizing purified antibodies onto an agarose support. After being washed and elutioned, the immunoprecipitates were subjected to western blot assays.

### Biotin switch assay for *S*-nitrosylation

Detection of *S*-nitrosylated proteins was performed using a kit (Cat. No. 10006518, Cayman Chemical) that employs a modification of the previously described ‘biotin-switch’ method.^43^ Using this method, nitrosyl-groups are replaced with biotin residues. Biotinylated proteins were then precipitated by western blotting.

### Immunohistochemistry evaluation

Tumor tissues embedded in paraffin from both the control and L-NAME-treated groups were collected from previous experiment of our group. And 4 *μ*M thick tissue sections were prepared on slides and then were dewaxed and rehydrated in xylene and graded alcohols. After antigen retrieval, slides were blocked with 0.3% hydrogen peroxide (Zhongshan Gold Bridge, Beijing, China) and 5% BSA, followed by primary antibody at 4 °C overnight. The indicated antibody was incubated according to the manufacturer’s protocol. After washing with PBS, the slides were incubated with secondary antibody (mouse/rabbit probe HRP labeling) for 30 min at room temperature and then loaded onto DAB mixed reagent for 5 min. Counterstain of hematoxylin was applied for 3 min.

### MTT assay

Cells per well were plated overnight in 96-well plate. After treatment with indicated compounds for the indicated time period, 10 *μ*l MTT (5 mg/ml) was added in each well. After incubated for 2–4 h at 37 °C when the purple precipitate is clearly visible, 100 *μ*l of DMSO was added to all wells including controls. Swirl gently for 15 min and record absorbance at 490 nm by BioTek Instruments (Winooski, VT, USA).

### Apoptosis assay

The percentage of cells undergoing apoptosis was determined using Annexin V-FITC/PI double staining with the Apoptosis Detection Kit (KeyGEN BioTECH, Nanjing, China). The assay was performed according to the manufacturer’s instructions. Briefly, after performing the indicated treatments, cells were collected and washed twice with pre-cold PBS buffer, and then cells were stained with Annexin V and PI in 1×binding buffer. The percentage of cells undergoing apoptosis was determined using FACSCalibur (BD Biosciences, Franklin lakes, NJ, USA).

### Statistical analysis

Each experiment was repeated at least three times. For all the quantitative analyses represented in the histograms, the values are expressed as the mean values±S.D. The significance of the differences between mean values were assessed using Student’s *t*-test. All computations were calculated using the SPSS 20.0.

## References

[bib1] Kroemer G , Mariño G , Levine B . Autophagy and the integrated stress response. Mol Cell 2010; 40: 280–293. 20965422 10.1016/j.molcel.2010.09.023 PMC3127250

[bib2] Murrow L , Debnath J . Autophagy as a stress-response and quality-control mechanism: implications for cell injury and human disease. Annu Rev Pathol 2013; 8: 105–137. 23072311 10.1146/annurev-pathol-020712-163918 PMC3971121

[bib3] Notte A , Leclere L , Michiels C . Autophagy as a mediator of chemotherapy-induced cell death in cancer. Biochem Pharmacol 2011; 82: 427–434. 21704023 10.1016/j.bcp.2011.06.015

[bib4] Doria A , Gatto M , Punzi L . Autophagy in human health and disease. N Engl J Med 2013; 368: 1845. 10.1056/NEJMc1303158 23656659

[bib5] Fulda S , Kögel D . Cell death by autophagy: emerging molecular mechanisms and implications for cancer therapy. Oncogene 2015; 34: 5105–5113. 25619832 10.1038/onc.2014.458

[bib6] Knowles RG , Moncada S . Nitric oxide synthases in mammals. Biochem J 1994; 298: 249–258. 7510950 10.1042/bj2980249 PMC1137932

[bib7] Thomas DD , Ridnour LA , Isenberg JS , Flores-Santana W , Switzer CH , Donzelli S et al. The chemical biology of nitric oxide: implications in cellular signaling. Free Radic Biol Med 2008; 45: 18–31. 18439435 10.1016/j.freeradbiomed.2008.03.020 PMC2572721

[bib8] Sarkar S , Korolchuk VI , Renna M , Imarisio S , Fleming A , Williams A et al. Complex inhibitory effects of nitric oxide on autophagy. Mol Cell 2011; 43: 19–32. 21726807 10.1016/j.molcel.2011.04.029 PMC3149661

[bib9] Tripathi DN , Chowdhury R , Trudel LJ , Tee AR , Slack RS , Walker CL et al. Reactive nitrogen species regulate autophagy through ATM-AMPK-TSC2-mediated suppression of mTORC1. Proc Natl Acad Sci USA 2013; 110: E2950–E2957. 23878245 10.1073/pnas.1307736110 PMC3740898

[bib10] Haldar SM , Stamler JS . S-nitrosylation at the interface of autophagy and disease. Mol Cell 2011; 43: 1–3. 21726803 10.1016/j.molcel.2011.06.014

[bib11] Xu W , Liu LZ , Loizidou M , Ahmed M , Charles IG . The role of nitric oxide in cancer. Cell Res 2002; 12: 311–320. 12528889 10.1038/sj.cr.7290133

[bib12] Fukumura D , Kashiwagi S , Jain RK . The role of nitric oxide in tumour progression. Nat Rev Cancer 2006; 6: 521–534. 16794635 10.1038/nrc1910

[bib13] Yang Z , Klionsky DJ . Mammalian autophagy: core molecular machinery and signaling regulation. Curr Opin Cell Biol 2010; 22: 124–131. 20034776 10.1016/j.ceb.2009.11.014 PMC2854249

[bib14] He C , Klionsky DJ . Regulation mechanisms and signaling pathways of autophagy. Annu Rev Genet 2009; 43: 67–93. 19653858 10.1146/annurev-genet-102808-114910 PMC2831538

[bib15] Yuan TL , Cantley LC . PI3K pathway alterations in cancer: variations on a theme. Oncogene 2008; 27: 5497–5510. 18794884 10.1038/onc.2008.245 PMC3398461

[bib16] Hafsi S , Pezzino FM , Candido S , Ligresti G , Spandidos DA , Soua Z et al. Gene alterations in the PI3K/PTEN/AKT pathway as a mechanism of drug-resistance (review). Int J Oncol 2012; 40: 639–644. 22200790 10.3892/ijo.2011.1312

[bib17] Numajiri N , Takasawa K , Nishiya T , Tanaka H , Ohno K , Hayakawa W et al. On-off system for PI3-kinase-Akt signaling through S-nitrosylation of phosphatase with sequence homology to tensin (PTEN). Proc Natl Acad Sci USA 2011; 108: 10349–10354. 21646525 10.1073/pnas.1103503108 PMC3121815

[bib18] Wang HM , Wu XY , Xia YF , Qian JY . Expression of ATM protein in nasopharyngeal carcinoma cell lines with different radiosensitivity. Ai Zheng 2003; 22: 579–581. 12948404

[bib19] Li J , Fan Y , Chen J , Yao KT , Huang ZX . Microarray analysis of differentially expressed genes between nasopharyngeal carcinoma cell lines 5-8F and 6-10B. Cancer Genet Cytogenet 2010; 196: 23–30. 19963132 10.1016/j.cancergencyto.2009.08.004

[bib20] Wei L , Gravitt PE , Song H , Maldonado AM , Ozbun MA . Nitric oxide induces early viral transcription coincident with increased DNA damage and mutation rates in humanpapillomavirus-infected cells. Cancer Res 2009; 69: 4878–4884. 19487298 10.1158/0008-5472.CAN-08-4695 PMC3820841

[bib21] Zhou L , Zhu DY . Neuronal nitric oxide synthase: structure, subcellular localization, regulation, and clinical implications. Nitric Oxide 2009; 20: 223–230. 19298861 10.1016/j.niox.2009.03.001

[bib22] Avontuur JA , Tutein Nolthenius RP , Buijk SL , Kanhai KJ , Bruining HA . Effect of L-NAME, an inhibitor of nitric oxide synthesis, on cardiopulmonary function in human septic shock. Chest 1998; 113: 1640–1646. 9631805 10.1378/chest.113.6.1640

[bib23] Tang CH , Grimm EA . Depletion of endogenous nitric oxide enhances cisplatin-induced apoptosis in a p53-dependent manner in melanoma cell lines. J Biol Chem 2004; 279: 288–298. 14576150 10.1074/jbc.M310821200

[bib24] Wang W , Wen Q , Xu L , Xie G , Li J , Luo J et al. Activation of Akt/mTOR pathway is associated with poor prognosis of nasopharyngeal cancer. PLoS One 2014; 9: e106098. 25165983 10.1371/journal.pone.0106098 PMC4148345

[bib25] Shang L , Wang X . AMPK and mTOR coordinate the regulation of Ulk1 and mammalian autophagy initiation. Autophagy 2011; 7: 924–926. 21521945 10.4161/auto.7.8.15860

[bib26] Hillier BJ , Christopherson KS , Prehoda KE , Bredt DS , Lim WA . Unexpected modes of PDZ domain scaffolding revealed by structure of nNOS-syntrophin complex. Science 1999; 284: 812–815. 10221915

[bib27] Saur D , Paehge H , Schusdziarra V , Allescher HD . Distinct expression of splice variants of neuronal nitric oxide synthase in the human gastrointestinal tract. Gastroenterology 2000; 118: 849–858. 10784584 10.1016/s0016-5085(00)70171-5

[bib28] Ikenoue T , Inoki K , Zhao B , Guan KL . PTEN acetylation modulates its interaction with PDZ domain. Cancer Res 2008; 68: 6908–6912. 18757404 10.1158/0008-5472.CAN-08-1107

[bib29] Wu H , Feng W , Chen J , Chan LN , Huang S , Zhang M . PDZ domains of Par-3 as potential phosphoinositide signaling integrators. Mol Cell 2007; 28: 886–898. 18082612 10.1016/j.molcel.2007.10.028

[bib30] Song MS , Salmena L , Pandolfi PP . The functions and regulation of the PTEN tumour suppressor. Nat Rev Mol Cell Biol 2012; 13: 283–296. 22473468 10.1038/nrm3330

[bib31] Trotman LC , Alimonti A , Scaglioni PP , Koutcher JA , Cordon-Cardo C , Pandolfi PP . Identification of a tumour suppressor network opposing nuclear Akt function. Nature 2006; 441: 523–537. 16680151 10.1038/nature04809 PMC1976603

[bib32] Lee AW , Ma BB , Ng WT , Chan AT . Management of nasopharyngeal carcinoma: current practice and future perspective. J Clin Oncol 2015; 33: 3356–3364. 26351355 10.1200/JCO.2015.60.9347

[bib33] Wang L , Li G , Yao ZQ , Moorman JP , Ning S . MicroRNA regulation of viral immunity, latency, and carcinogenesis of selected tumor viruses and HIV. Rev Med Virol 2015; 25: 320–341. 26258805 10.1002/rmv.1850

[bib34] Chalhoub N , Baker SJ . PTEN and the PI3-kinase pathway in cancer. Annu Rev Pathol 2009; 4: 127–150. 18767981 10.1146/annurev.pathol.4.110807.092311 PMC2710138

[bib35] Lim HJ , Crowe P , Yang JL . Current clinical regulation of PI3K/PTEN/Akt/mTOR signalling in treatment of human cancer. J Cancer Res Clin Oncol 2015; 141: 671–689. 25146530 10.1007/s00432-014-1803-3 PMC11823746

[bib36] Ciuffreda L , Falcone I , Incani UC , Del Curatolo A , Conciatori F , Matteoni S et al. PTEN expression and function in adult cancer stem cells and prospects for therapeutic targeting. Adv Biol Regul 2014; 56: 66–80. 25088603 10.1016/j.jbior.2014.07.002

[bib37] Hopkins BD , Hodakoski C , Barrows D , Mense SM , Parsons RE . PTEN function: the long and the short of it. Trends Biochem Sci 2014; 39: 183–190. 24656806 10.1016/j.tibs.2014.02.006 PMC4043120

[bib38] Wang Z . Protein S-nitrosylation and cancer. Cancer Lett 2012; 320: 123–129. 22425962 10.1016/j.canlet.2012.03.009

[bib39] Monteiro HP , Costa PE , Reis AK , Stern A . Nitric oxide: protein tyrosine phosphorylation and protein S-nitrosylation in cancer. Biomed J 2015; 38: 380–388. 26068128 10.4103/2319-4170.158624

[bib40] Kwak YD , Ma T , Diao S , Zhang X , Chen Y , Hsu J et al. NO signaling and S-nitrosylation regulate PTEN inhibition in neurodegeneration. Mol Neurodegener 2010; 5: 49. 21067594 10.1186/1750-1326-5-49 PMC2992530

[bib41] Christopherson KS , Hillier BJ , Lim WA , Bredt DS . PSD-95 assembles a ternary complex with the N-methyl-D-aspartic acid receptor and a bivalent neuronal NO synthase PDZ domain. J Biol Chem 1999; 274: 27467–27473. 10488080 10.1074/jbc.274.39.27467

[bib42] Sugi T , Oyama T , Morikawa K , Jingami H . Structural insights into the PIP2 recognition by syntenin-1 PDZ domain. Biochem Biophys Res Commun 2008; 366: 373–378. 18062914 10.1016/j.bbrc.2007.11.138

[bib43] Jaffrey SR , Snyder SH . The biotin switch method for the detection of S-nitrosylated proteins. Sci STKE 2001; 2001: pl1. 10.1126/stke.2001.86.pl1 11752655

