# Publisher Correction: NOS1 *S*-nitrosylates PTEN and inhibits autophagy in nasopharyngeal carcinoma cells

**DOI:** 10.1038/s41419-019-1476-6

**Published:** 2019-03-25

**Authors:** Lingqun Zhu, Linlin Li, Qianbing Zhang, Xiao Yang, Zhiwei Zou, Bingtao Hao, Francesco M Marincola, Zhengjun Liu, Zhuo Zhong, Meng Wang, Xiaoxuan Li, Qianli Wang, Keyi Li, Wenwen Gao, Kaitai Yao, Qiuzhen Liu

**Affiliations:** 1 Cancer Research Institute, Southern Medical University, Guangzhou, China; 2 Sidra Medical and Research Center, Out-Patient Clinic, PO Box 26999, Doha, Qatar; 3 Department of Vascular Surgery, Nanfang Hospital Southern Medical University, Guangzhou, China

**Correction to:** **Cell Death & Disease**

10.1038/cddiscovery.2017.11;

published online 20 February 2017.

It was brought to the attention of the Editors that there had been an accidental duplication of the western blot images during the preparation of Figs. 1b and c. The authors were notified about the error and have supplied the correct image for Fig. 1c (below). We apologize for any inconvenience this may have caused the readers.

**Fig. 1:**
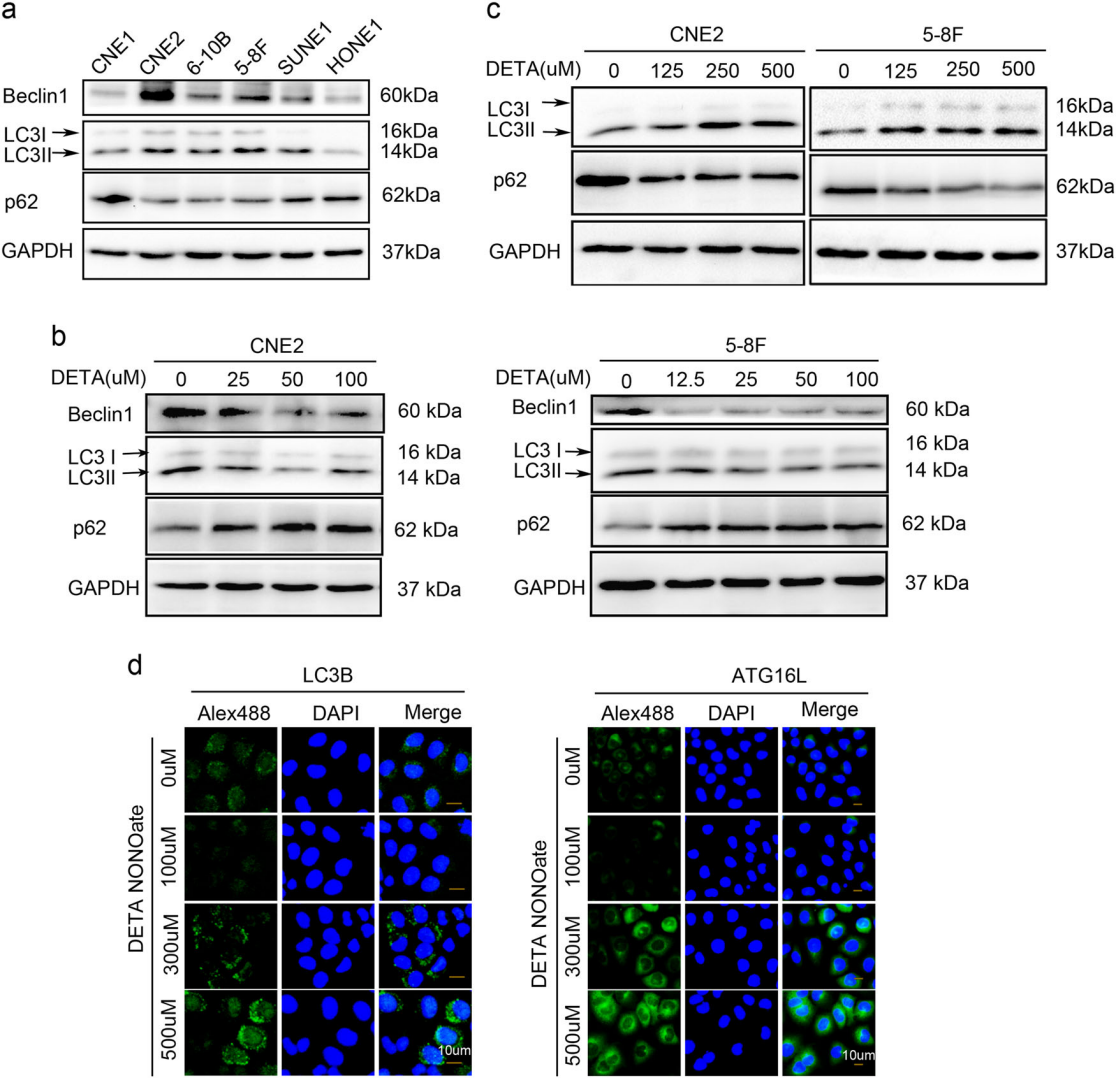
Exogenous NO plays a dual role in regulation of autophagy. **a** Basic level of autophagy in six of NPC cell lines was tested by western blot using antibodies against LC3B, Beclin1 (autophagy-related protein) and p62 (autophagy substrate). **b** Immunoblot shows that autophagy was inhibited by low concentration of exogenous NO (DETA-NONOate 0–100 μM for 24 h) in CNE2 (left) and 5–8F (right). **c** Immunoblot shows that autophagy was increased by high concentration of exogenous NO (DETA NONOate 125–500 μM for 24 h) in CNE2 (left) and 5–8F (right). **d** Representative images by inversed fluorescent microscope showed that LC3B (left) and ATG16L1 (right) were inhibited by low dose but increased by high dose of NO after immunostained with primary antibodies and corresponding FITC-conjugated secondary antibodies. Nuclei were counterstained with DAPI. Representative images of each sample are shown. (Blots were probed for GAPDH as a control for equal protein loading in all lanes)

This has been corrected in both the PDF and HTML versions of the Article.

